# Effect of Different Display Types on Vection and Its Interaction With Motion Direction and Field Dependence

**DOI:** 10.1177/2041669517707768

**Published:** 2017-05-05

**Authors:** Behrang Keshavarz, Martina Speck, Bruce Haycock, Stefan Berti

**Affiliations:** Toronto Rehabilitation Institute – University Health Network (UHN), iDAPT, Toronto, ON, Canada; Department of Psychology, Ryerson University, Toronto, ON, Canada; Department of Psychology, Johannes-Gutenberg University Mainz, Mainz, Germany; Toronto Rehabilitation Institute – University Health Network (UHN), iDAPT, Toronto, ON, Canada; University of Toronto, Institute for Aerospace Studies, Toronto, ON, Canada; Department of Psychology, Johannes-Gutenberg University Mainz, Mainz, Germany

**Keywords:** illusory self-motion, field of view, optic flow, circular vection, field dependence or field independence, cognitive style, visual stimulation, perception

## Abstract

Illusory self-motion (vection) can be generated by visual stimulation. The purpose of the present study was to compare behavioral vection measures including intensity ratings, duration, and onset time across different visual display types. Participants were exposed to a pattern of alternating black-and-white horizontal or vertical bars that moved either in vertical or horizontal direction, respectively. Stimuli were presented on four types of displays in randomized order: (a) large field of view dome projection, (b) combination of three computer screens, (c) single computer screen, (d) large field of view flat projection screen. A Computer Rod and Frame Test was used to measure field dependence, a cognitive style indicating the person’s tendency to rely on external cues (i.e., field dependent) or internal cues (i.e., field independent) with respect to the perception of one’s body position in space. Results revealed that all four displays successfully generated at least moderately strong vection. However, shortest vection onset, longest vection duration, and strongest vection intensity showed for the dome projection and the combination of three screens. This effect was further pronounced in field independent participants, indicating that field dependence can alter vection.

## Introduction

Vection typically describes the sensation of illusory self-motion in the absence of physical movement (Brandt, Dichgans, & Koenig, 1973; Fischer & Kornmueller, 1930; Hettinger, Schmidt, Jones, & Keshavarz, 2014). A classic situation in which vection occurs is sitting in a stationary train waiting for departure from the station. When an adjacent train starts moving, passengers on the stationary train often get the feeling of self-motion. This sensation of self-motion is perceived in the opposite direction of the moving train. Similar vection sensations can be frequently experienced in driving or flight simulators, virtual environments, or large-screen movie theaters (e.g., Palmisano, Allison, Schira, & Barry, 2015). Vection intensity can vary depending on several factors. For instance, vection has been reported to be facilitated when redundant multisensory information is provided, such as when auditory cues are added to a visual stimulus (Keshavarz, Hettinger, Vena, & Campos, 2014; Riecke, Väljamäe, & Schulte-Pelkum, 2009). Other factors that can determine vection intensity include stimulus speed (e.g., Tamada & Seno, 2015) or stimulus density (e.g., Lubeck, Bos, & Stins, 2015). In this study, we will investigated the role of further factors that could affect vection on a technical, sensory, and individual level. That is, we varied (a) the visual display type (technical level), (b) stimulus’ motion direction (sensory level), and (c) field dependence as cognitive style (individual level), with the overall goal to determine optimal settings for investigating vection in laboratory research. In the following, we will first describe these three potential influencing factors in detail, before summarizing our hypotheses.

### Visual Display Types

Strong sensations of vection can be easily generated using stimulation of the entire visual field, for instance, in optokinetic rotating drums (e.g., Brandt, Dichgans, & Koenig, 1972) or tumbling and swinging rooms (e.g., Allison, Howard, & Zacher, 1999). On the other hand, it is not well understood how the experience of vection is affected by different display types that cover varying amounts of the central and peripheral visual field, such as computer screens, virtual reality glasses, or large projection screens, under otherwise identical conditions (e.g., stimulus type, stimulus speed, stimulus size, spatial frequency etc.). A recent study by Riecke and Jordan (2015) compared subjective vection ratings for visual stimuli presented on three stereoscopic devices, including a three-dimensional (3D) TV, a projection screen, and a head mounted display. The size of the field-of-view (FOV) was held constant across the displays. Results showed no differences in vection ratings between the different display types. However, most of the current laboratory research investigating vection often uses rather simple, nonstereoscopic presentation settings such as standard computer screens to generate vection. As previous research demonstrated that reducing the size of the FOV hampers vection (e.g., Allison et al., 1999; Dichgans & Brandt, 1978; Flanagan, May, & Dobie, 2002; Riecke & Jordan, 2015), it has been questioned to which extent these simple settings are actually capable of inducing vection instead of suggesting object motion (Palmisano et al., 2015), and how compelling the sensation of vection is on these simple displays compared with more realistic and complex laboratory settings. In other words, are simple computer screens appropriate tools for vection research? To answer this question, we presented the same stimulus—a visual pattern of alternating black-and-white vertical or horizontal bars that has been previously shown to successfully induce circular vection in a variety of laboratory settings (Dichgans & Brandt, 1978; Hu et al., 1997; Keshavarz & Berti, 2014; Palmisano & Gillam, 1998; Webb & Griffin, 2002)—on four different displays, ranging from a very basic setting with a small FOV (single computer monitor) to a technically demanding setup including multiple projectors with a large FOV (dome projection). The dome projection setting covered the entire horizontal visual field and a large proportion of the vertical visual field, comparable to a classic optokinetic rotating drum. Thus, we considered the dome projection setup as our “gold standard” for generating vection. The other three displays were compared with this gold standard with regard to vection intensity, vection duration, and vection onset time (i.e., the first moment that vection is perceived. Consequently, the main outcome of the present study would be an evaluation of the degree to which a simple technical solution for inducing vection can be compared with a more realistic setup (i.e., our gold standard) in laboratory research.

### Motion direction

The second factor that was varied was the direction of motion of the stimulus. In one condition, vertical bars moved horizontally to the right or left, whereas in the other condition, horizontal bars moved vertically up or down. The purpose of this manipulation was to test whether there was a difference in vection perception between horizontal and vertical movement with similar stimuli. For instance, Kano (1991) exposed his participants to peripheral visual stimulation that included horizontal or vertical stimulus movement and measured subjective vection ratings. Results showed that vection onset time was shorter for stimulus movement in the vertical (i.e., up-down) direction compared with movement in the horizontal direction (i.e., right–left). Similar results were reported by Telford and Frost (1993), who compared six different motion directions including left, right, up, and down movement. Again, vection was perceived faster and was more intense during vertical motion compared with horizontal motion. In the present study, we were particularly interested in interactions between stimulus motion direction (vertical vs. horizontal) and different display types.

### Field Dependence

Perceptual processing can vary between individuals (see, for instance, Witkin et al., 1954). One well-established phenomenon in vision is so-called field dependence (e.g., Bendall, Galpin, Marrow, & Cassidy, 2016; Witkin & Goodenough, 1977), a personality trait that has been demonstrated by a number of studies to tap a basic cognitive style which affects different aspects of perceptual processing (see Boccia, Piccardi, Marco, Pizzamiglio, & Guariglia, 2016). Field dependence describes the tendency to which extent the perception of one’s body position in space is influenced by contextual information. That is, field dependent persons rely stronger on external cues (e.g., reference frame, background objects), whereas field independent persons rely stronger on internal cues (e.g., vestibular, proprioceptive) for perception of body position. The concept of field dependence has been previously applied in several contexts, such as spatial navigation (e.g., Boccia et al., 2016) or postural responses. For instance, field dependent persons were found to show decreased postural stability compared to field independent persons during a sharpened Romberg test in darkness (e.g., Isableu, Ohlmann, Cremieux, & Amblard, 1997). Additionally, the sensation of simulator sickness was reported to be higher in field independent participants than in field dependent participants (Barrett & Thornton, 1968). Field dependence has also been associated with behavioral responses that are not limited to perceptual processing, such as social behavior (often referred to as cognitive style, see Witkin & Goodenough, 1977). These findings suggest that it is generally important to consider interindividual variability in the processing of sensory information. Interindividual differences in the degree of field dependence in visual sensory processing might be especially relevant in the context of the present study because different vection-inducing displays with different perceptual frames will be tested with regard to vection. It seems plausible that field dependence might affect the perception of vection in situations where a visual frame is missing and is not clearly indicative of the Earth’s vertical. This is the case when no screen frame is visible, for instance, in situations where the entire visual field is stimulated. Therefore, field dependence was added as a third factor in order to unravel a potential effect of this cognitive style on the perception of vection and potential interactions with the display type.

### Research Questions and Hypotheses

Vection is a multifaceted phenomenon and, as such, factors at various levels can affect the subjective perception of it. In the present study, we aimed to investigate three factors contributing to vection that involve technical (i.e., presentation settings), sensory (i.e., visual stimulus motion direction), and individual (i.e., field dependence) variations.

In detail, the main goal of this study was to test whether vection can be reliably induced using simple visual displays commonly used in vection research. Additionally, we aimed to find out how intense the sensation of vection (if present at all) is under such simple settings compared with highly immersive and compelling visual displays. We also manipulated the stimulus’ visual motion direction and considered the individual level of field dependence to account for potential interactions between display type and individual and sensory factors. Participants were thus exposed to a visual pattern of horizontally or vertically moving bars for several seconds. The task of the participants was to rate vection in a two-step procedure. First, they were instructed to indicate whether they experienced vection by indicating immediately when vection occurred during the visual stimulation (i.e., vection onset time). Second, participants were asked after each stimulus to rate vection intensity and duration for each trial.

In particular, we hypothesized that (a) vection is experienced in all of the four display settings but that vection is most compelling in the dome projection screen due to the large, curved FOV, and the immersive scene. In more detail, we expected strongest vection intensity, fastest vection onset time, and longest vection duration within the dome presentation compared with the other three display types. Based on previous findings, (b) we also expected that vection measures are affected by the manipulation of the stimulus features, with stronger vection induced by vertical motion patterns. Finally, (c) with respect to field dependence, we had no directed hypothesis but rather wanted to explore whether this personality trait might have an effect on the perception of vection.

## Methods

### Participants

Thirteen male (*M*_age_ = 26.62 years, range: 18–44 years) and seventeen female (*M*_age_ = 27.06 years, range: 20–45 years) healthy adults (no recent history of stroke, active vestibular disorders, disabling musculoskeletal disorder, acute psychiatric disorder, and a diagnosis of dementia or mild cognitive impairment) participated voluntarily in this study; no reward was given after completion of the study. All participants had normal or corrected-to-normal vision and were naive with respect to the purpose of the study. The study protocol was approved by the Toronto Rehabilitation Institute’s Research Ethics Board and was conducted according to the guidelines of the Declaration of Helsinki. Written informed consent was obtained prior to the beginning of the study and participants were instructed that they could stop the experiment at any time without negative consequences; however, no participant decided to abort the experiment prematurely.

### Stimuli and Apparatus

**Figure 1.**
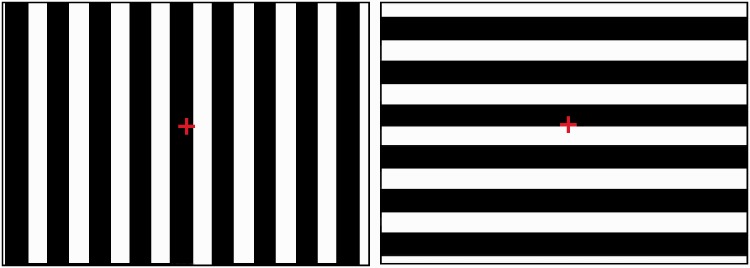
Stimulus pattern consisting of vertical (left panel) and horizontal (right panel) altered black-and-white stripes. The vertical bars move to the right/left and the horizontal bars move up/down. A static fixation cross (red) is superimposed to the center of the screen.

Visual stimulation consisted of patterns of alternating black-and-white vertical or horizontal stripes of uniform thickness (see Figure 1), with an average spatial frequency of 0.13 cycles/degree for each display. That is, a single combination of black-and-white bars covered the same portion of the visual field in each display, or, in other words, the stimulus size was constant across all displays. For the monitors and simple projector with two-dimensional rendering, the stimuli moved in four different directions: the vertical bars moved either to the right or to the left and the horizontal bars moved either upwards or downwards, resulting in four trials per display. For the dome projection, which uses a 3D rendering environment, the stripes are a uniform 0.13 cycles/degree, forming a striped cylinder that rotates in yaw or pitch. The duration of each trial was 45 s.^1^ Stimulus’ speed was held constant across all trials and all displays and was set to 1 cycle/s. In addition to the moving stimuli, a static red cross was presented in the center of the stimulus (see Figure 1); this cross served as a fixation cross during stimulus presentation.

**Figure 2.**
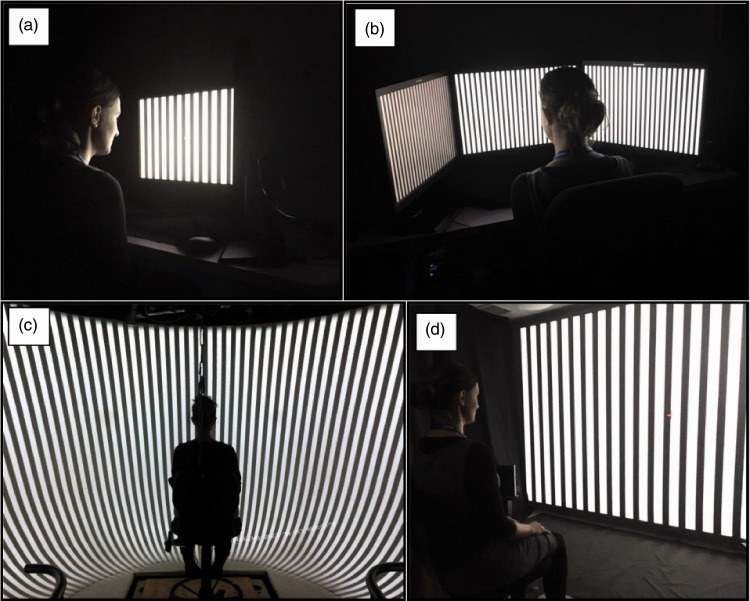
Laboratory setup for each of the four display conditions, including single screen (a), three screens (b), StreetLab (c), and projector (d).

The main variation of the study was that four different display types were used to present the stimuli (see Figure 2 and Table 1 for an overview): (a) StreetLab: StreetLab is a dome-shaped virtual reality laboratory and is part of the Challenging Environment Assessment Center at the Toronto Rehabilitation Institute. Six projectors are used to create a seamless, curved projection screen. The refresh rate was 60 Hz and the display resolution was 1920 × 1200 pixels for each projector. (b) Three screens: Array of three 24″ ThinkVision TFT monitors aligned next to each other (approx. angle of 120° between monitors). The refresh rate was 60 Hz and display resolution was 1920 × 1200 pixels for each monitor. (3) Single screen: 24″ ThinkVision TFT monitor with a refresh rate of 60 Hz and a display resolution of 1920 × 1200 pixels. (4) Projector: A large projection screen (300 cm × 196 cm) with Optoma HD 850 projector, refresh rate of 60 Hz, and a display resolution of 1920 × 1080 pixels. In all display conditions, participants were seated in a height-adjustable chair with eye-height leveled to the center of the screen. Each of the eight trials was presented once on each display type in randomized order. In general, the stimuli were presented in temperature controlled, dimly-lit rooms without windows.

**Table 1.** Overview and Details of the Four Display Types.

	StreetLab	Three screens	Single screen	Projector
Specifications	Dome projection	3 × 24″ TFT monitors	24″ TFT monitor	Flat projection
User distance	150 cm	26 cm	26 cm	200 cm
Field-of-view (horizontal × vertical)	240° × 105°	220° × 52°	78° × 52°	78° × 52°
Spatial frequency	0.13 cycles/ degree	0.13 cycles/ degree	0.13 cycles/ degree	0.13 cycles/ degree
Stimulus speed	1 cycle/s	1 cycle/s	1 cycle/s	1 cycle/s

### Design

#### Independent measures

Each stimulus type was presented once on each display. The order of trial presentation was randomized for each display and the sequence of display order was randomized as well; direction of movement was merged to horizontal (left and right) and vertical (upwards, downwards) movement.^2^ This resulted in a 4 × 2 × 2 design, including the within-subjects factors display type (StreetLab vs. three screens vs. single screen vs. projector) and motion direction (horizontal movement vs. vertical movement), as well as the between-subjects factor field dependence (low vs. high).

#### Dependent measures

Participants were instructed to report different subjective measures, that including vection onset time, vection intensity, vection duration, direction of vection, as well as motion sickness. (a) Vection onset time was defined as the time between onset of the visual stimulation and the verbally indicated onset of perceived vection by the participant. (b) Vection intensity was indicated after each trial using a verbal rating scale ranging from 0 (*no vection at all*) to 10 (*intense vection*). (c) Vection duration was estimated by the participant after each trial, indicating the proportion of time that vection was experienced (in percent). In addition to these common measures of vection, the participants also reported (d) the direction of vection they experienced (*left, right, up, down*), and (e) potential adverse side-effects (i.e., incidences of motion sickness) which were assessed via the Fast Motion Sickness Scale (FMS, Keshavarz & Hecht, 2011), a verbal, subjective measurement of nausea and stomach awareness on a scale ranging from 0 (*no sickness*) to 20 (*severe sickness*). Note that no participants reported motion sickness (average FMS scores < 1).

### Procedure

Participants first underwent a prescreening to ensure that they were eligible to participate in the study. Visual acuity was assessed using a Snellen Test which all participants passed (i.e., scores better than 20/30). Prior to the actual study, participants were asked to fill out questionnaires measuring individual anxiety (STAI, Spielberger & Sydeman, 1994), depersonalization (Sierra & Berrios, 2000), and motion sickness susceptibility (Golding, 2006).

**Figure 3.**
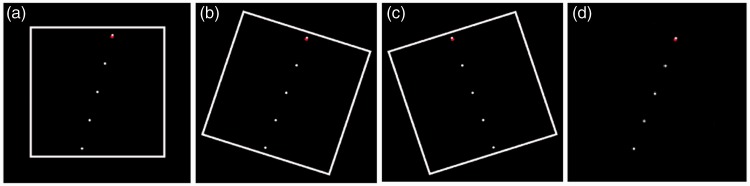
The four configurations of the Computer Rod and Frame Test (CRAF): (a) nontilted frame, (b) frame tilted 18°clockwise, (c) frame tilted 18° counter-clockwise, and (d) no frame.

Before stimulus presentation, participants also completed a computer-based version of the Rod and Frame Test (CRAF; Bagust, Rix, & Hurst, 2005) as a measure of field dependence. The Rod and Frame Test, originally developed by Witkin et al. (1954), provides a quantitative measure of the subjective perception of verticality and is a widely accepted tool to measure the cognitive style of field dependence. The CRAF consists of a vertical, luminescent rod (consisting of five linearly aligned dots) surrounded by a luminescent frame (see Figure 3). The participant’s task is to align the rod with respect to the Earth’s vertical using the left and right button of a computer mouse. The surrounding frame is either stable or tilted clockwise (18°) or counter-clockwise (18°). Errors in the alignment of the rod (measured in degrees) represent deviations from the true vertical. The rationale of the CRAF is that participants with high field dependence are more affected by the surrounding frame than those with low field dependence. In other words, smaller errors in the alignment of the rod indicate a low level of field dependence because these participants are less affected by the surrounding frame but rely more on their internal cues that give rise to subjective verticality (e.g., vestibular and proprioceptive information). The CRAF was presented on a large projection screen (300 cm × 196 cm) with the visual frame size of 187 × 187 cm, resulting in a FOV of 50.1° horizontally and vertically. Participants were seated on a height-adjustable chair with their eye-height leveled to the center of the screen. To remove any visual cues to verticality, the CRAF was conducted in the dark. Participants had to wear a customized pair of glasses (no lens strength) that limited the FOV to the projection screen and covered the peripheral visual field. Participants had to vertically align the rod four times in each of the four conditions (no frame, frame not tilted, frame tilted clockwise 18°, and frame tilted counter-clockwise 18°). Deviations from the true vertical position were measured in degrees. A positive score indicated a drift of the aligned rod in counter-clockwise, whereas a negative score indicated a drift of the rod in clockwise direction. An average score for each of the four trials per condition was generated and used for statistical analyses.

All participants were then exposed to all experimental trials which included each of the four displays. The order of displays was counterbalanced and the order of trials within each display was randomized. Vection onset time was defined as the time between onset of the visual stimulation and the verbally indicated onset of perceived vection by the participant; this time was manually measured by the experimenter. Ratings on vection intensity, vection duration, and FMS were recorded after each trial. A short rest break was provided between each display condition and, additionally, participants were free to have a break between each trial, if desired. To minimize eye movement during stimulus presentation, participants were instructed to direct their eyes on the red fixation cross (12 mm × 12 mm) presented in the center of the screen.

## Results

Mixed-factorial repeated-measures analysis of variance (rmANOVAs) including the within-subjects factors display (StreetLab, three screens, single screen, projector) and motion direction (horizontal, vertical) and the between-subjects factor field dependence (low, high) were computed for the vection measures onset time, intensity, and duration. A median split of the averaged absolute error (i.e., deviation from the true vertical in degrees) across all CRAF trials (median = 1.34) was used to separate participants into two subgroups (*n* = 15 for each group), including those with high CRAF scores (i.e., high field dependence group; *M* = 2.45, *SD* = 0.94; 9 male, 6 female) and those with low CRAF scores (i.e., low field dependence group; *M* = 0.96, *SD* = 0.29; 8 male, 7 female).

### Vection Intensity

Averaged subjective ratings of vection intensity for each of the four displays is given in Table 2. One-sample *t* tests (against zero) showed that all four displays successfully induced vection (see Table 1). The rmANOVA revealed a significant main effect of display, *F*(3, 84) = 12.081, *p* < .001, $\eta_{p}^{2}$^ ^= .301. Post hoc comparisons (Bonferroni corrected) showed significantly stronger vection in StreetLab compared with the single screen (*p* < .001) and with the projector (*p* < .001). Similarly, the combination of three screens also resulted in higher vection intensity compared with the single screen (*p* = .015) and the projector (*p* = .010).

**Table 2.** Mean Vection Intensity Scores for Each Display Type and Results for One-Sampled *t* Tests (Against Zero).

Display	*M* (*SD*)	*t*	Lower 95% CI	Upper 95% CI
StreetLab	6.37 (1.70)	20.570***	5.73	7.00
Three screens	5.96 (1.81)	17.995***	5.28	6.64
Single screen	5.02 (1.66)	16.506***	4.40	5.64
Projector	4.73 (1.87)	13.833***	4.03	5.43

*Note.* All degrees of freedom = 29.

*** *p* < .001.

**Figure 4.**
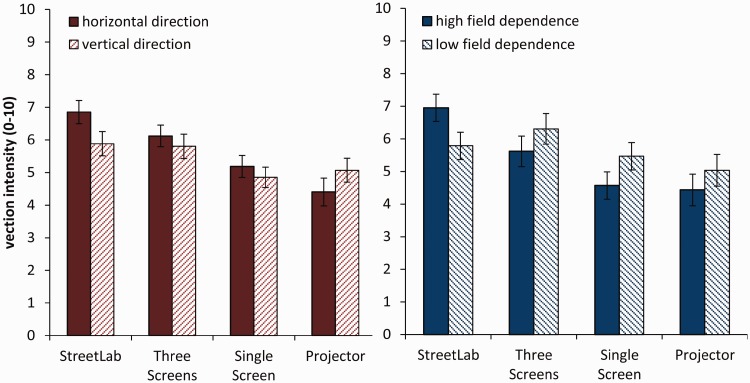
Average vection intensity ratings across the four display types separated by motion direction (left panel) and field dependence (right panel). Error bars indicate *SEM*.

Vection intensity ratings separated by field dependence (right panel) and motion direction (left panel) are shown in Figure 4. This indicates an interaction between display and direction which was also obtained by the rmANOVA, *F*(3, 84) = 4.099, *p* = .009, $\eta_{p}^{2}$^ ^= .128. Post hoc paired samples *t* tests (Bonferroni corrected) showed differences in vection intensity subject to motion direction in StreetLab, *t*(29) = 2.294, *p* = .029, Cohen’s *d* = 0.42, but not in the other three display types (*p*’s > .098, *d*’s < 0.31).

Additionally, an interaction between display and field dependence showed, *F*(3, 84) = 4.646, *p* = .005, $\eta_{p}^{2}$^ ^= .142. Separate rmANOVAs for each group showed differences in display type for the high field dependence group, *F*(3, 42) = 7.322, *p* < .001, $\eta_{p}^{2}$^ ^= .343, but not in the low field dependence group, *F*(3, 42) = 0.860, *p* = .470, $\eta_{p}^{2}$^ ^= .058. That is, in the high field dependence group, StreetLab generated significantly stronger vection compared with the projector (*p* = .023) and the single screen (*p* = .033). No other main effect or interaction was found.

### Vection Onset Time

**Figure 5.**
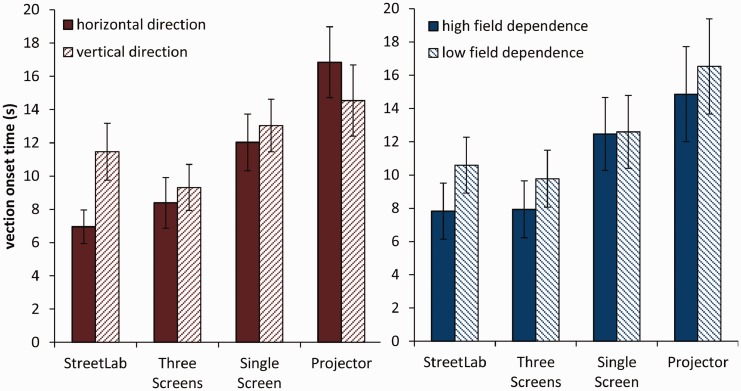
Average vection onset time across the four display types (left), separated by motion direction (middle) and field dependence (right). Error bars indicate *SEM*.

Averaged subjective vection onset time for each of the four displays is given in Table 3. The mean vection onset times for the four displays separated by field dependence and motion direction is shown in Figure 5. One-sample *t* tests (against 45 s) showed that vection was perceived before the end of each 45 s-long trial across all four displays (see Table 2). The rmANOVA revealed a significant main effect of display, *F*(3, 84) = 10.049, *p* < .001, $\eta_{p}^{2}$^ ^= .264. Post hoc comparisons showed significantly prolonged vection onset times for the projector compared with StreetLab (*p* = .002) and the three screens condition (*p* = .001).

**Table 3.** Mean Vection Onset Times in Seconds for Each Display Type and Results for One-Sampled *t* Tests (Against 45).

Display	*M* (*SD*)	*t*	Lower 95% CI	Upper 95% CI
StreetLab	9.21 (6.55)	29.938***	6.76	11.65
Three screens	8.86 (6.57)	30.123***	6.40	11.31
Single screen	12.53 (8.34)	21.317***	9.42	15.65
Projector	15.69 (10.92)	14.696***	11.61	19.77

*Note.* All degrees of freedom = 29.

*** *p* < .001.

An interaction between display and direction showed as well, *F*(3, 84) = 4.267, *p* = .007, $\eta_{p}^{2}$^ ^= .132, indicating that differences in vection onset time between the four displays were more prominent when the bars moved in horizontal direction than in vertical direction. Post hoc paired sampled *t* tests (Bonferroni corrected) showed differences in vection onset time subjected to motion direction in StreetLab, *t*(29) = 3.002, *p* = .005, *d* = 0.55, but not in the other three display types (*p*’s > .119, *d*’s < 0.29). No other main effect or interaction was found.

### Vection Duration

**Figure 6.**
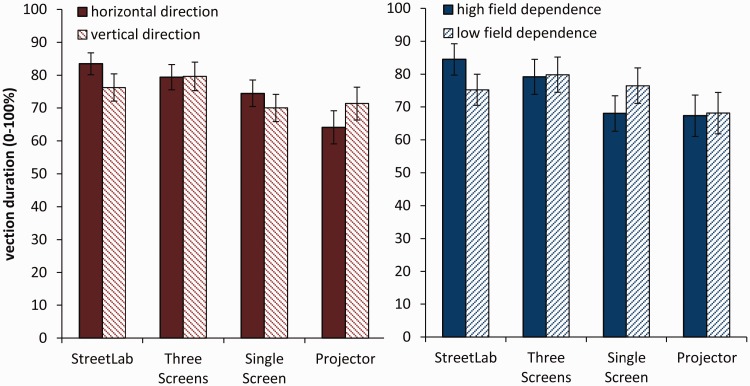
Average vection duration across the four display types (left), separated by motion direction (middle) and field dependence (right). Error bars indicate SEM.

Averaged subjective ratings of vection duration for each of the four displays is given in Table 4. Vection duration ratings separated by field dependence and motion direction are shown in Figure 6. One-sample *t* tests (against zero) showed that all four displays successfully induced vection (see Table 3). The rmANOVA revealed a significant main effect of display, *F*(3, 84) = 5.026, *p* = .003, $\eta_{p}^{2}$^ ^= .152. Post hoc comparisons showed significantly shorter vection duration for the projector compared with StreetLab (*p* = .049) and the three screens condition (*p* = .048).

**Table 4.** Mean Vection Duration in Percent for Each Display Type and Results for One-Sampled *t* Tests (Against zero).

Display	*M* (*SD*)	*t*	Lower 95% CI	Upper 95% CI
Street Lab	79.87 (18.74)	23.348***	72.87	86.86
Three Screens	79.53 (20.32)	21.435***	71.94	87.11
Single Screen	72.25 (21.14)	18.721***	64.36	80.14
Projector	67.73 (24.13)	15.375***	58.72	76.73

*Note.* All degrees of freedom = 29.

*** *p* < .001.

An interaction between display and direction (see Figure 5, left) was also obtained, *F*(3, 84) = 3.501, *p* = .019, $\eta_{p}^{2}$^ ^= .111, indicating that differences in vection duration between the four displays varied due to motion direction. Post hoc paired samples *t* tests (Bonferroni corrected) showed differences in vection duration subject to motion direction in StreetLab, *t*(29) = 2.185, *p* = .037, *d* = 0.40, unlike the other three display types (*p*’s > .128, *d*’s < 0.28). No other main effect or interaction was found.

## Discussion

The goal of this study was to compare the subjective sensation of vection across different display types for stimuli that moved in different directions. Further, we were interested in whether vection is modulated by the individual level of field dependence. As expected, our results showed that StreetLab and the three screens display setting generated stronger vection intensity, shorter vection onset times, and longer vection duration compared with the single screen and projector setting. In addition, we found that field dependence affected vection: The difference in displays was more pronounced in participants who showed high field independency. We will discuss these findings in detail in the following sections.

### Display Type

All of the four display types used in the present study generated at least moderate vection. As expected, the dome projection (i.e., StreetLab as “gold standard”) elicited strongest vection with longest duration and shortest onset times and outscored both the single screen and the projector in all of the vection ratings. However, the combination of three screens turned out to be comparably successful in generating vection, suggesting that a laboratory setup that consists of an array of computer screens with simplified stimuli is also very efficient for conducting basic vection research (see also Nakamura, 2010). In contrast to StreetLab and the three screens combination, the single screen setup resulted in significantly decreased vection ratings. Nonetheless, vection still occurred frequently and was perceived as moderately intense in this condition supporting the assumption that a single screen is indeed sufficient to induce a moderate level of vection (e.g., Keshavarz & Berti, 2014).

The discrepancy in vection ratings between the different display types might be best explained by the size of the visual FOV. That is, the two displays with the largest FOV—StreetLab and the combination of three screens—resulted in strongest vection ratings overall. Previous research found similar results, showing that a smaller FOV typically reduces the sensation of vection (e.g., Allison et al., 1999; Dichgans & Brandt, 1978; Flanagan et al., 2002; Riecke & Jordan, 2015). The role of visual stimulation of the peripheral and central FOV has been exhaustively discussed in this context (e.g., Berthoz, Pavard, & Young, 1975; Warren & Kurtz, 1992).

In addition, it could be possible that the vection difference between the displays is due to the nature of the vection percept in each of the displays. Stimulus presentation in the projection dome arguably created circular vection along the yaw axis, but it remains speculative whether this is also true for the remaining three display settings due to their flat and noncurved nature. One could assume that the three monitors also generated circular vection due to the angular alignment of the screens covering a good portion of the peripheral visual field. In the single screen and projector condition, however, this seems questionable. Although we did not ask all participants whether they experienced circular or linear vection in each of the displays, anecdotal reports after the experiment indicated that some participants indeed felt circular vection in the single screen condition, whereas others felt linear vection.^3^ Regardless of the nature of vection perceived in each display, our results suggest that vection was less compelling and less strong in the single monitor and projector setting. As mentioned before, this finding needs to be confirmed with other stimuli, particularly with those inducing noncircular vection (e.g., radially expanding stimuli inducing vection along the fore-and-aft axis).

In contrast to the FOV, the shape of the projection screen (i.e., curved vs. straight) did not affect vection ratings: Both StreetLab and the linearly aligned array of three screens revealed similar vection ratings. However, it remains to be answered whether this effect is consistent across different stimuli types. In this study, we used rather artificial stimuli to generate vection in general and circular vection in particular. It is possible that scenarios that exhibit a higher level of reality and other types of vection (e.g., linear vection in the fore-and-aft direction) might elicit stronger vection in a dome projection display. Note that stimulus speed and stimulus size were held constant across the different display settings by varying the distance between the observer and the displays. The absolute distance from the display is unlikely to affect the perception of vection (see Nakamura, 2006), however, other factors, such as luminance, contrast, or accommodation of the eyes, were not controlled for. We cannot disregard a potential influence of these factors on the vection ratings.

### Motion Direction

An interaction between display type and motion direction was found across all vection ratings. In each case, vection ratings varied between horizontally and vertically moving stimuli in StreetLab only. In detail, horizontally moving bars resulted in stronger vection intensity, longer vection duration, and shorter vection onset times compared with vertically moving bars. Differences in vection perception between horizontally and vertically moving stimuli have been previously investigated with mixed results. For instance, Kano (1991; see also Telford & Frost, 1993) provided his participants with peripheral stimulation of the visual system by having a monitor positioned to the left and right side of their head, whereas the center of the visual field was filled by a gray cardboard. Results showed stronger vection for vertically moving stimuli compared with horizontally moving stimuli. In contrast, Nakamura and Shimojo (2000) exposed their participants to a visual stimulus that contained simultaneous motion of the background and the foreground. The authors asked their participants to focus exclusively on the foreground motion: No differences in vection ratings were found when the foreground moved horizontally or vertically.

The difference in vection ratings between horizontal and vertical stimulus motion in StreetLab could be explained by the nature of StreetLab and the corresponding perception of moving stimuli. Due to StreetLab’s dome-shaped environment with 3D rendering, the bar stimuli created the sensation of rotation about the yaw or pitch axis, the latter of which generating a feeling of “falling over.” In contrast, in the other displays, up and down motion of the stimuli were not necessarily perceived as rotation about the pitch axis but rather as linear up-and-down movement, comparable to being in an elevator. The difference in motion direction could be due to the perception of circular pitch rotation, which—in contrast to translational movement and rotation along the yaw axis—conflicts with the body’s vertical axis. Consequently, a full pitch rotation along 360° might be perceived as more unlikely than a full yaw rotation, in which the vertical axis is still aligned with gravity. Also, note that the effect in StreetLab itself is not strong per convention (Cohen’s *d* indicating medium effect size; Cohen, 1977). Given that the effect of motion direction was missing in three of our four display types, one may conclude that the direction of the movement (i.e., horizontal vs. vertical) is, in general, not a crucial factor in vection perception. However, this has to be evaluated in more detail in further studies.

### Field Dependence

The concept of field dependence describes to what extent a person perceives an object independent from its surrounding. In other words, the perception of one’s body position is not influenced by the contextual surrounding (e.g., a visual frame) in high field independent observers. The present study is the first to investigate a potential role of field dependence on the sensation of vection. Our results demonstrated differences in vection measures between participants with high and low field dependence in StreetLab but not in any of the other three display types. This finding can potentially be explained by the outer frame of the different visual displays used in this study: In those settings in which a frame surrounding the visual screen was present and easily detectable (i.e., due to the edges of the screens or the projector), the frame provided distinct visual information about the position of the observer’s body indicating a vertical, upright position. Although one could assume that field independent individuals are more likely to experience stronger vection in these display settings because they are less affected by their surrounding environment, our findings did not support this assumption. This could be due to the fact that the information regarding the true vertical delivered by the reference frame of the screen was too dominant and overruled the effect of field independence in this context. In contrast, in settings in which the reference frame was missing or at least minimized (i.e., StreetLab), this distinct external cue regarding body position was missing and participants had to rely more on internal cues. Consequently, as field independent observers generally rely more on internal body cues (e.g., vestibular, proprioceptive) rather than on external background cues, it is plausible they experienced less vection compared with field dependent observers because their internal cues suggest stasis. The correlation between field dependence and vection also points to the relevance of a reference frame in perceptual processing in general. For instance, Telford and Frost (1993) reported that vection was absent in their study when a frame was occluded, and Keshavarz, Hecht, and Zschutschke (2011) demonstrated that simulator sickness is increased when a stationary background surrounds the visual stimulus. These findings suggest that information regarding the visual surrounding is automatically integrated when processing visually induced motion sensations.

The role of external frames has also been discussed in the context of spatial navigation, where participants either use an allocentric or an egocentric reference frame for ongoing integration of changes in the sensory input due to locomotion (see, for instance, Goeke, König, & Gramann, 2013). This distinction describes whether people either navigate with reference to objects in space (i.e., allocentric reference frame) or with reference to their own orientation in space (i.e., egocentric reference frame; see Klatzky, 1998). A common, underlying foundation of the two concepts of field dependence and ego- or allocentric reference frames is likely; however, it needs to be further determined whether these sources of interindividual differences interact with each other in the context of perceived self-motion and vection. At this point, it remains speculative that field dependent participants rely on a reference frame to asses self-motion on basis of purely visual information because there are no further studies reporting a direct link between field dependence and vection. Future studies may test the relation between vection, spatial orientation, and field dependence in more detail. In addition, the integration of additional personality measures to vection research seems fruitful in order to evaluate further factors modulating the individual degree of perceived vection.

## Summary and Conclusion

In the present study, the perception of self-motion (i.e., vection) was elicited by all four display settings, ranging from rather simple (e.g., single computer screen) to highly complex (e.g., dome projection) display types. This finding has several important implications. First, our results are in line with previous studies demonstrating that participants can perceive vection (although less intense) under simple laboratory settings with comparably basic or coarse visual motion stimuli. In other words, vection can be generated using a single monitor screen, although the experience is less intense and less reliable. Second, our findings demonstrate that the combination of three screens is highly effective in generating compelling vection and is a simple setup to test vection in laboratory research. This raises the question whether the three screen setup could be established as a new “gold standard” of visual stimulus presentation for basic vection research in order to facilitate the integration and comparability of results in vection research over different laboratories. Finally, the perception of vection was modulated by interindividual variations of field dependence, suggesting that this cognitive style is a factor that should be generally considered in vection research.

## Notes

1 Note that in addition to this stimulation, there were also four trials with horizontally or vertically moving visual stimuli with higher spatial frequency (i.e., 20 cycles/degree), but due to technical problems (i.e., different speeds of motion), data analysis was confined to the four stimuli with the lower spatial frequency because these stimuli were comparable to the stimulation in our previous study (Keshavarz & Berti, 2014).

2 Repeated-measures ANOVAs revealed no differences in any of the vection ratings between right versus left and up versus down motion of the bars, respectively. Thus, the four motion directions were merged into the two motion categories horizontal (left and right) and vertical (up and down).

3 This finding was confirmed by a brief follow-up study testing five subjects in the single screen and three screens conditions with horizontal motion. Results showed that all participants experienced circular vection in the three screens condition, and that 60% of the subjects reported circular vection in the single screen condition, confirming that the bar stimulus may be ambiguous with respect to the type of vection generated.
